# Risk factors for severe and prolonged cough in influenza and COVID-19: A *post hoc* analysis of three randomized controlled trials

**DOI:** 10.1016/j.pccm.2025.11.007

**Published:** 2025-12-12

**Authors:** Yang Jin, Qidan Hu, Jingya Li, Dong Liu, Xiaoying Gu, Jiuyang Xu, Lian Liu, Honglin Hu, Wei Wang, Yeming Wang, Bin Cao

**Affiliations:** a School of Medicine, Tsinghua Medicine, Tsinghua University, Beijing 100084, China; b Capital Medical University, National Center for Respiratory Medicine, State Key Laboratory of Respiratory Health and Multimorbidity, National Clinical Research Center for Respiratory Diseases, Institute of Respiratory Medicine, Chinese Academy of Medical Sciences, Department of Pulmonary and Critical Care Medicine, Center of Respiratory Medicine, China-Japan Friendship Hospital, Beijing 100029, China; c Department of Clinical Statistics and Data Management, Jiangsu Simcere Pharmaceutical, Nanjing, Jiangsu 210042, China; d Tsinghua University-Peking University Joint Center for Life Sciences, Beijing 100084, China

To the Editor,

Cough is an essential protective reflex that facilitates the clearance of airway secretions and foreign material. Among the many etiologies of cough, respiratory virus infections (RVIs) represent the most frequent cause globally.^1^ Although cough is often regarded as a nonspecific and self-limited symptom, marked heterogeneity exists in its severity and duration across individuals. In a subset of patients, cough persists beyond the period of acute illness, resulting in postinfectious cough (PIC), which is clinically defined as a subacute cough lasting 3–8 weeks following RVIs.^2^

This study used previously collected data from clinical trials that had received ethical approval by the ethics committee at China-Japan Friendship Hospital (with the approval number of 2020-118-Y33, YW2022-021-02 and YW2022-017-01 respectively for the phase 2, 3 influenza trials and phase 2–3 COVID-19 trial) and each other center. Written informed consent was obtained from adult participants. For adolescent participants, written parental consent was obtained. The data were de-identified, and the analysis does not involve new interventions or new data collected from participants. We confirm that no additional ethical approval is required for this secondary analysis.

In this *post hoc* analysis, we examined data from three randomized controlled trials that investigated new antiviral therapies for acute uncomplicated influenza or mild-to-moderate coronavirus disease 2019 (COVID-19). The primary aim was to identify risk factors associated with severe or prolonged cough during RVIs, with the goal of informing early clinical intervention strategies for at-risk populations.

For influenza, we analyzed data from phase 2 and phase 3 clinical trials evaluating suraxavir marboxil (GP681) (ClinicalTrials.gov identifiers: NCT04736758 and NCT05474755).^3^^,^^4^ For COVID-19, we assessed data from a phase 2–3 trial evaluating oral simnotrelvir (SIM0417) co-administered with ritonavir in patients with mild-to-moderate disease (NCT05506176).^5^ Only individuals with laboratory-confirmed infection based on reverse transcription polymerase chain reaction (RT-PCR) were included (Fig. 1A). To maximize statistical power, patients in both the antiviral and placebo groups were included.

**Fig. 1:**
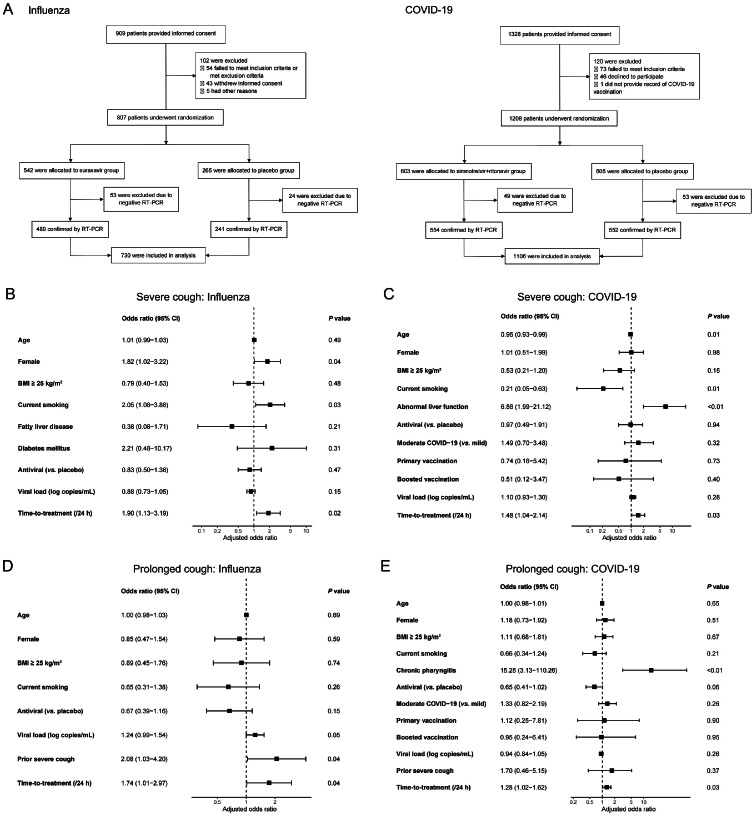
(A) Flow diagram illustrating inclusion and exclusion of the study population. (B and C) Forest plots of multivariable logistic regression showing risk factors associated with severe cough in (B) influenza and (C) COVID-19. (D and E) Forest plots showing risk factors associated with prolonged cough in (D) influenza and (E) COVID-19. All models were adjusted for the variables presented. BMI: Body mass index; CI: Confidence interval; COVID-19: Coronavirus disease 2019; RT-PCR: Reverse transcription polymerase chain reaction.

Baseline demographic and clinical characteristics were collected, including age, sex, body mass index (BMI), smoking status, comorbidities, treatment assignment (antiviral or placebo), time-to-treatment (defined as the interval between symptom onset and first dose of study drug or placebo), baseline viral load, COVID-19 severity, and COVID-19 vaccination status. Across all three trials, symptoms were recorded at baseline (day 1) and on days 2, 3, 5, 7, and 15 after treatment initiation. Seven symptoms were consistently assessed, including cough, fever or chills, nasal congestion or rhinorrhea, sore throat, fatigue, headache, and muscle or joint pain. Symptom severity was self-reported using a standardized 4-point scale (0 indicating absence of symptoms, 1 mild, 2 moderate, and 3 severe, Supplementary Tables 1 and 2). Mild cough was defined as intermittent coughing without impairment of daily activities or work. Severe cough was defined as frequent or intense coughing during the day and night that interfered with daily activities, work, or sleep. Moderate cough was defined as intermediate severity. Patients were classified as having severe cough if they reported a score of 3 at any time from baseline through follow-up. Prolonged cough was defined as the presence of any cough symptom (score ≥1) persisting through the end of the 2-week observation period.

Categorical variables were summarized as frequencies with percentages, and continuous variables as medians with interquartile ranges (IQR). Between-group comparisons were performed using Mann–Whitney *U* tests for continuous variables and chi-squared tests or Fisher’s exact tests for categorical variables, as appropriate. Multivariable logistic regression analysis was conducted to identify independent predictors of severe or prolonged cough, adjusting for all relevant demographic and clinical covariates when applicable. For the influenza data, a mixed-effects logistic regression model incorporating a random intercept for trial phase was used to account for potential differences between the phase 2 and phase 3 trials, and trial-by-predictor interaction effects were examined and excluded. Comorbidities that did not reach statistical significance in univariable analyses (*P* > 0.05) were excluded from multivariable logistic regression analysis to avoid overfitting. Multicollinearity among variables was evaluated using the variance inflation factor. Two complementary sensitivity analyses were performed: (1) bootstrap resampling with 1000 replicates to generate 95 % bias-corrected and accelerated (BCa) confidence intervals (CIs), and (2) repeat multivariable logistic regression analysis after excluding participants with underlying respiratory diseases, including chronic obstructive pulmonary disease (COPD), asthma, chronic rhinitis, chronic pharyngitis, and chronic cough. To assess whether the association between time-to-treatment and cough outcomes differed by treatment modality, an interaction term between treatment groups and time-to-treatment was included in the multivariable logistic regression models, and subgroup analyses were performed. All statistical analyses were performed using R software (version 4.3.0; R Foundation for Statistical Computing, Vienna, Austria). A *P* value <0.05 was considered statistically significant.

In total, 730 patients with influenza and 1106 with COVID-19 were included in this analysis (Fig. 1A). Among participants with influenza, the median age was 28.0 (IQR: 21.0–36.0) years, and 316 (43.3 %) were women (Supplementary Table 3). The median time-to-treatment was 26.3 (IQR: 18.5–36.9) h. Most individuals were nonsmokers (79.9 %, 583/730), and the most common comorbid conditions were chronic rhinitis (*n* = 49, 6.7 %), fatty liver disease (*n* = 44, 6.0 %), and hyperuricemia (*n* = 37, 5.1 %). Among patients with COVID-19, the median age was 35.0 (IQR: 28.0–47.0) years, and 457 (41.3 %) were women. The median time-to-treatment was 47.9 (IQR: 31.8–62.3) h. Similar to the influenza cohort, most were nonsmokers (78.8 %, 872/1106). The most common comorbidities were hypertension (*n* = 88, 8.0 %), hyperuricemia (*n* = 73, 6.6 %), and fatty liver disease (*n* = 68, 6.1 %). The majority of COVID-19 cases were classified as moderate illness (*n* = 718, 64.9 %), and most participants had received a booster dose of the COVID-19 vaccine (*n* = 854, 77.2 %).

At baseline, 88.2 % (*n* = 644) of patients with influenza and 72.2 % (*n* = 799) of those with COVID-19 reported cough, and the prevalence of the other six symptoms assessed was similarly high (Supplementary Fig. 1). Although all symptoms declined over the course of illness, cough remained the most frequently reported symptom from Day 2 onward, indicating slower recovery compared with other symptoms. In the influenza cohort, cough resolved within 1 week in 49.5 % (361/730) and within 2 weeks in 85.4 % (593/694) of individuals. Similarly, in the COVID-19 cohort, resolution occurred within 1 week in 50.4 % (557/1106) and within 2 weeks in 83.0 % (875/1054) of individuals. By day 15, residual cough persisted in 14.6 % (101/694) and 17.0 % (179/1054) of patients with influenza and COVID-19, respectively (Supplementary Table 4). Severe cough (defined as a maximum symptom score of 3 at any follow-up visit) occurred in 15.5 % (113/730) of patients with influenza and 7.6 % (84/1106) of those with COVID-19 (Supplementary Table 5), demonstrating a significantly higher frequency in influenza (*P* < 0.001). Nearly all cases of unresolved cough at day 15 were classified as mild (Supplementary Fig. 1).

Multivariable logistic regression demonstrated that, in influenza, female sex (adjusted odds ratio [aOR] 1.82, 95 % CI: 1.02–3.22), current smoking (aOR 2.05, 95 % CI: 1.08–3.88), and prolonged time-to-treatment (per 24 h, aOR 1.90, 95 % CI: 1.13–3.19) were independently associated with severe cough (Fig. 1B). In COVID-19, younger age (aOR 0.96, 95 % CI: 0.93–0.99), current smoking (aOR 0.21, 95 % CI: 0.05–0.63), abnormal liver function (aOR 6.86, 95 % CI: 1.99–21.12), and prolonged time-to-treatment (aOR 1.48, 95 % CI: 1.04–2.14) were significantly associated with severe cough (Fig. 1C). All risk factors remained robust across sensitivity analyses (Supplementary Tables 6–7).

Prolonged time-to-treatment also emerged as an independent risk factor for prolonged cough in both influenza (aOR 1.74, 95 % CI: 1.01–2.97) and COVID-19 (aOR 1.28, 95 % CI 1.02–1.62) (Fig. 1D and E), and these associations persisted in sensitivity analyses (Supplementary Tables 8–9). A history of severe cough (aOR 2.08, 95 % CI: 1.03–4.20) was associated with prolonged cough in influenza, though this result was not significant in bootstrap analysis. Chronic pharyngitis (aOR 15.25, 95 % CI: 3.13–110.26) was associated with prolonged cough in COVID-19. Antiviral therapy demonstrated a nonsignificant trend toward lower risk of prolonged cough in both influenza (aOR 0.67, 95 % CI: 0.39–1.16, *P* = 0.15) and COVID-19 (aOR 0.65, 95 % CI: 0.41–1.02, *P* = 0.06).

In treatment-specific multivariable logistic regression analysis, the associations between delayed time-to-treatment and either severe or prolonged cough were similar across antiviral and placebo groups. For severe cough, the aOR (95 % CI) corresponding to each 24-hour delay was 1.90 (0.99–3.71) in the antiviral group and 2.01 (0.80–5.03) in the placebo group for influenza, and 1.52 (0.94–2.49) and 1.46 (0.85–2.54), respectively, for COVID-19 (Supplementary Table 10). For prolonged cough, the corresponding aOR (95 % CI) was 1.72 (0.88–3.36) versus 1.95 (0.75–5.09) in influenza, and 1.36 (1.02–1.82) versus 1.18 (0.85–1.64) in COVID-19. Although underpowered due to reduced sample size, the subgroup analyses consistently demonstrated that effect estimates were directionally congruent with the primary analysis. In addition, no significant interaction was observed between treatment group and time-to-treatment (*P* > 0.05). Therefore, these further analyses reinforced that delayed therapeutic initiation was associated with more severe and longer-lasting cough, regardless of antiviral use.

In this study, cough emerged as the most prevalent symptom and demonstrated the slowest rate of resolution, thereby contributing substantially to delayed overall symptom recovery. Approximately 50 % of patients experienced resolution of cough within 1 week and approximately 85 % within 2 weeks across both influenza and COVID-19 cohorts, whereas only around 15 % continued to report cough beyond 2 weeks. In a subset of individuals, cough persisted for several weeks after the acute illness phase, a condition known as PIC.^2^ Although the pathogenesis of PIC remains incompletely understood, disruption and inflammation of the respiratory mucosa, increased mucus hypersecretion, and heightened cough reflex sensitivity have been proposed as key mechanisms.^2^ Further mechanistic studies are warranted to elucidate biological pathways and host factors that predispose certain patients to PIC.

Although most risk factors identified in this study differed between influenza and COVID-19, delayed time-to-treatment was consistently associated with both severe and prolonged cough in both diseases. These findings suggest that earlier therapeutic intervention may mitigate cough severity and duration in RVIs. Notably, the association between treatment delay and cough outcomes was independent of treatment modality (antiviral *vs*. placebo), as demonstrated by subgroup and interaction analyses. For patients receiving antiviral therapy, improved outcomes are biologically plausible given the enhanced efficacy of antiviral agents when administered early. The benefit associated with early treatment among placebo recipients is less readily explained but may reflect placebo-related effects previously described in clinical trials of cough suppressants.^1^ Collectively, these observations support the potential role of timely clinical management to prevent severe and persistent cough during RVIs. Although the underlying mechanism remains unclear, reduced inflammatory burden or attenuation of cough reflex sensitivity may be contributing factors. Additionally, antiviral therapy exhibited a non-significant trend toward reducing prolonged cough compared with placebo in both disease groups, which may reflect insufficient power to detect a treatment effect.

Severe cough occurred more frequently in influenza than in COVID-19. This difference in clinical severity likely reflects intrinsic pathogen characteristics. Most COVID-19 cases included in this analysis were attributable to the Omicron variant, which has shown lower pathogenicity relative to earlier severe acute respiratory syndrome coronavirus 2 (SARS-CoV-2) variants. Moreover, influenza viruses preferentially bind sialic acid α2,6-galactose receptors, which are abundantly expressed in the upper airway, whereas angiotensin-converting enzyme 2 (ACE2) receptors required for SARS-CoV-2 entry are expressed at relatively low levels in the upper respiratory tract. Further research is needed to clarify the distinct mechanisms underlying cough severity in different RVIs.

This study has several limitations. First, as a *post hoc* analysis of completed clinical trials, the findings are subject to potential bias. Second, the moderate sample size limited statistical power, particularly for comorbidities such as COPD, gastroesophageal reflux disease (GERD), and chronic cough, preventing robust evaluation of their associations with cough outcomes. Third, the follow-up duration was insufficient to assess PIC, which is defined as a cough lasting 3–8 weeks after acute illness. Finally, the study population consisted exclusively of individuals with nonsevere influenza or COVID-19, limiting the generalizability of the findings to severe disease. Future studies incorporating larger and more heterogeneous cohorts, longer follow-up, objective cough measurements, immune profiling, and the development of risk prediction models are needed to better understand the underlying mechanisms and clinical heterogeneity of PIC. The recognition of “long COVID”^6^ and similar post-viral syndromes like “long flu” necessitates research into whether non-resolving symptoms, such as cough, share a common etiology and underlying pathogenic mechanisms.

In conclusion, this *post hoc* analysis of three RCTs demonstrated that delayed time-to-treatment was consistently associated with both severe and prolonged cough in influenza and COVID-19, independent of antiviral or placebo therapy, emphasizing the clinical importance of early intervention.

## Funding

This study was supported by Beijing Municipal Health Commission Research Ward Excellence Clinical Research Program (No. BRWEP2024W114060100), 10.13039/501100005090Beijing Nova Program (No. 20230484343), 10.13039/501100005150Chinese Academy of Medical Sciences (CAMS) Innovation Fund for Medical Sciences (No. 2023-I2M-C&T-B-119), and New Cornerstone Science Foundation to B.C.

## CRediT authorship contribution statement

**Yang Jin:** Writing – original draft, Visualization, Validation, Software, Methodology, Investigation, Formal analysis, Data curation. **Qidan Hu:** Writing – original draft, Investigation, Formal analysis, Data curation. **Jingya Li:** Validation, Methodology, Investigation. **Dong Liu:** Validation, Resources, Methodology, Investigation. **Xiaoying Gu:** Validation, Supervision, Methodology, Investigation. **Jiuyang Xu:** Validation, Supervision, Methodology, Investigation. **Lian Liu:** Resources, Project administration, Data curation. **Honglin Hu:** Resources, Project administration, Data curation. **Wei Wang:** Resources, Data curation. **Yeming Wang:** Writing – review & editing, Supervision, Resources, Project administration, Conceptualization. **Bin Cao:** Writing – review & editing, Supervision, Resources, Project administration, Funding acquisition, Conceptualization.

## Declaration of competing interest

The author Bin Cao is the Associate Editor for this journal and was not involved in the editorial review or the decision to publish this article. B.C. serves as a steering committee member for the baloxavir transmission study, sponsored by F. Hoffmann-La Roche Ltd; he also acts as the Principal Investigator for the GP681 trial in outpatients with influenza, sponsored by Jiangxi Qingfeng Pharma Group. All other authors report no conflicts of interest.

## Appendix Supplementary materials

Image, application 1
