# Supplementary material for: Risk factors for severe and prolonged cough in influenza and COVID-19: A post hoc analysis of three randomized controlled trials
Source: Chin Med J Pulm Crit Care Med. 2025 Dec 12;3(4):319–22. doi: 10.1016/j.pccm.2025.11.007 (PMC12805390; doi:10.1016/j.pccm.2025.11.007)
Supplement: Supplementary file 1 [file mmc1.docx]

**Supplementary Material**

**Inclusion and exclusion criteria for the original** **clinical trials**

For influenza, data were obtained from phase 2 and phase 3 randomized controlled trials evaluating suraxavir marboxil (GP681) for the treatment of acute uncomplicated influenza (ClinicalTrials.gov identifiers NCT04736758 and NCT05474755).^1,2^ The inclusion criteria for the phase 2 trial were: (1) age 18–65 years; (2) fever with an axillary temperature ≥37.3℃; (3) a positive rapid antigen test for influenza; and (4) at least one systemic and one respiratory symptom of influenza with a severity of at least moderate intensity within 2 days of symptom onset. The phase 3 trial employed similar criteria but additionally included adolescents aged 5–18 years. Patients requiring hospitalization or with concomitant bacterial infections or non-influenza viral infections (including SARS-CoV-2) were excluded.

For COVID-19, data were derived from a phase 2 to 3 trial investigating orally co-administered simnotrelvir (SIM0417) and ritonavir in adults with mild-to-moderate COVID-19 (ClinicalTrials.gov identifier NCT05506176).^3^ Inclusion criteria were: (1) age ≥18 years; (2) onset of COVID-19 signs or symptoms within 3 days before the first study dose; (3) the presence of at least one COVID-19 symptom before the first dose of study drug or placebo; and (4) clinical presentation consistent with mild or moderate COVID-19 according to the U.S. Food and Drug Administration definition. Patients who required high-flow nasal cannula, noninvasive ventilation, invasive mechanical ventilation, or extracorporeal membranous oxygenation within 48 hours of screening, or those with serious renal, hepatic, or acute cardiovascular conditions, were excluded.


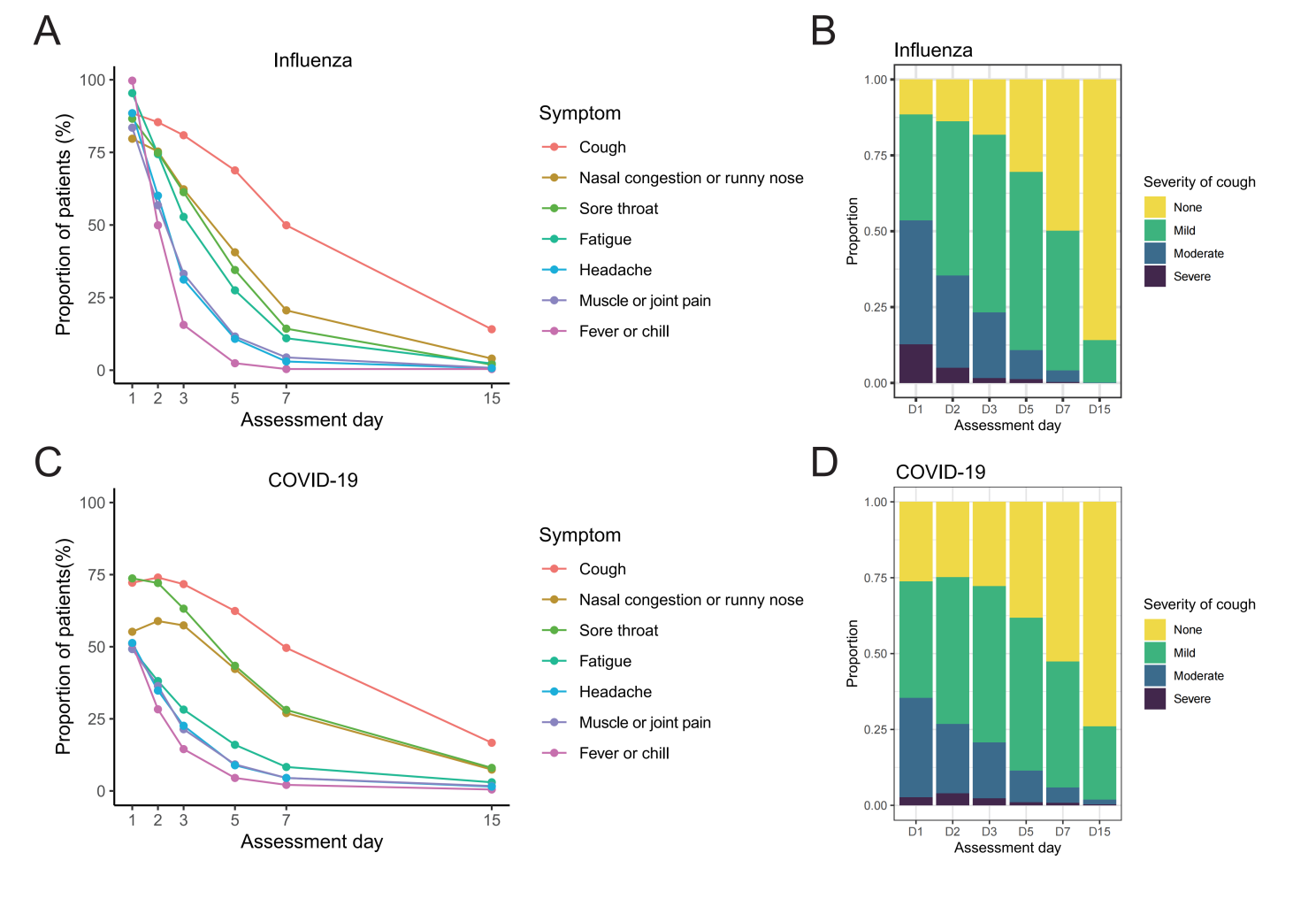


**Supplementary Fig. 1.** Prevalence and severity of cough at each assessment day. (A and C) Line chart showing the prevalence of the seven recorded symptoms at each assessment day in (A) influenza and (C) COVID-19. (B and D) Stacked bar plot showing the distribution of mild, moderate, and severe cough at each assessment day in (B) influenza and (D) COVID-19.

**Supplementary Table 1: Symptom assessment** **criteria in influenza: original trial score definitions.**

| **Influenza Symptoms** | **0** | **1** | **2** | **3** |
| --- | --- | --- | --- | --- |
| Cough | None | Mild: Intermittent cough does not affect daily life and work | Moderate: Between mild and severe | Severe: Frequent cough day and night, affecting life and work |
| Headache | None | Mild: Occasional pain | Moderate: Between mild and severe | Severe: Severe headache, affecting life and work |
| Feverishness or chills | None | Mild: Slight fever/chills | Moderate: obvious, but bearable | Severe: Feel obviously hot/cold, need to be covered warm |
| Muscle or joint pain | None | Mild: Occasionally, muscles and joints ache | Moderate: Between mild and severe | Severe: Severe muscle or joint pain, affecting life and work |
| Fatigue | None | Mild: Mild fatigue does not affect daily life and work | Moderate: Between mild and severe | Severe: Severe fatigue, affecting life and work |
| Nasal congestion | None | Mild: Slight nasal congestion on one side does not affect breathing | Moderate: Nasal congestion on both sides, lack of breathing | Severe: Bilateral nasal congestion is evident, requiring oral breathing |
| Sore throat | None | Mild: Occasional sore throat | Moderate: Between mild and severe | Severe: Severe sore throat, affecting life and work |

**Supplementary Table 2: Symptom assessment criteria in COVID-19: Original trial score definitions.**

|  | **Symptoms** | **Symptoms recovered before the** COVID**-19 Onset or Resolved (Score 0)** | **Mild (Score 1)** | **Moderate (Score 2)** | **Severe (Score 3)** |
| --- | --- | --- | --- | --- | --- |
| 1 | Cough | Occasional throat clearing, no irritating continuous cough, no discomfort; no impact on daily life. Or usually cough, but not aggravated after this COVID-19 infection. | Occasional transient coughing during the day, mildly affecting daily activities; or transient or occasional nocturnal coughing when falling asleep at night; mildly affecting sleep. | Intermittent cough during the day, more frequent and affecting daily activities, or sleep at night due to cough. | Frequent and intense coughing during the day, which severely impacts daily activities; inability to sleep at night due to coughing, or severely impacts sleep. |
| 2 | Stuffy or runny nose | No nasal congestion/running nose, or occasional slight nasal congestion/running nose, smooth ventilation; no effect on respiration, no effect on sleep at night. Or nasal congestion at ordinary times, but not aggravated after this COVID-19 infection. | Mild nasal congestion or runny nose; mild effects on respiration (slightly poor ventilation); or mild effects on sleep at night. | Significant nasal congestion or runny nose, affecting breathing (poor breathing), or sleep at night. | Severe nasal congestion or runny nose that severely impacts breathing (continuous lack of ventilation), inability to sleep at night, or severely impacts sleep. |
| 3 | Sore throat or dry throat | No throat pain or dryness; no significant discomfort with swallowing. Or usually dry throat, but not aggravated after this COVID-19 infection. | Mild sore throat or dry throat, mild discomfort when coughing or swallowing, relieved by drinking water. | Significant sore throat or dry throat, discomfort at rest, and need to drink plenty of water. | Severe sore throat or dry throat, persisting severe discomfort, unbearable, affecting swallowing, unrelieved by drinking water. |
| 4 | Shortness of breath or difficulty breathing | Daily activities (e.g., walking on flat ground) without chest distress or shortness of breath; or they appear only after a lot of exercise (e.g., heavy physical activities such as climbing stairs and moving heavy objects) and disappear after a short rest. Or there is usually this performance, but not aggravated after COVID-19 infection. | Shortness of breath (chest tightness, shortness of breath) after daily activities, which disappeared or was relieved after rest. | Shortness of breath (chest tightness, shortness of breath) below the usual activity amplitude and relieved by rest. | At rest (rest), shortness of breath (chest tightness, shortness of breath) or dyspnea is persistently felt and cannot be relieved. |
| 5 | Headache | No headaches, or occasional minor headaches; no impact on daily life. Or chronic headaches, not exacerbated by this COVID-19 infection. | Mild headache, tolerable, mildly affecting daily activities. | Moderate headache, long-lasting but tolerable, affecting daily activities. | Severe headache, unrelieved, intolerable, requiring rest. |
| 6 | Feeling hot or feverish | Ear temperature: <37.8°C  Oral temperature: <37.3°C  Axillary temperature: <37.0°C | Ear: 37.8–38.5°C  Oral: 37.3–38.0°C  Axillary: 37.0–37.7°C | Ear: 38.6–39.4°C  Oral: 38.1–38.9°C Axillary: 37.8–38.6°C | Ear: ≥39.5°C  Oral: ≥39°C  Axillary: ≥38.7°C |
| 7 | Chills or shivering (or cold intolerance) | No chills or shivering, no apparent cold discomfort. Or usually there is also fear of cold constitution, but not aggravated after this COVID-19 infection. | Mild chills or shivering confined to neck and (or) chest; Mild shaking; mild feeling of cold. | Moderate shivering or shaking, marked shaking of arms, neck and chest; marked feeling of coldness that requires clothing or heating to resolve. | Severe shivering or shaking, marked shaking of trunk and limbs; not relieved by the addition of clothing or heating. |
| 8 | Muscle or body aches (or soreness) | No pain, or only minor discomfort, no impact on daily life. or the presence of pain-related chronic diseases, which did not worsen after this COVID-19 infection. | Mild pain, tolerable, mild interference with daily activities. | Moderate pain, long-lasting but tolerable, affecting daily activities. | Severe pain, unrelieved, intolerable, requiring rest. |
| 9 | Nausea | No or transient mild nausea, no effect on food intake. | Intermittent nausea, mildly affecting the intake of food. | Frequent nausea leading to significant decrease in food intake. | Persistent nausea resulting in substantial reduction in food intake or inability to eat. |
| 10 | Vomit (throw up) | Stool character did not change significantly from usual, 0 times. | Record number of stools or watery stools  No vomit at all = 0  1–2 times= 1  3–4 times = 2  5 or more times = 3 | | |
| 11 | Diarrhea (loose or watery stools) | No vomiting, 0 episodes. | Number of vomiting recorded  No diarrhea at all = 0  1–2 times = 1  3–4 times = 2  5 or more times = 3 | | |

**Supplementary Table 3****: Baseline demographic and clinical characteristics of** **the study population.**

| Items | Influenza  （*N* =730） | COVID-19  （*N* = 1106） |
| --- | --- | --- |
| Age (years), median (IQR) | 28.0 (21.0–36.0) | 35.0 (28.0–47.0) |
| Female, *n* (%) | 316 (43.3) | 457 (41.3) |
| BMI category, *n* (%) |  |  |
| BMI <25 kg/m^2^ | 563 (77.1) | 713 (64.5) |
| BMI ≥25 kg/m^2^ | 167 (22.9) | 393 (35.5) |
| Smoking status, *n* (%) |  |  |
| Current smoker | 147 (20.1) | 234 (21.2) |
| Nonsmoker | 583 (79.9) | 872 (78.8) |
| Coronary heart disease, *n* (%) | 4 (0.5) | 0 |
| Hypertension, *n* (%) | 29 (4.0) | 88 (8.0) |
| Chronic lung disease, *n* (%) | 6 (0.8) | 34 (3.1) |
| COPD, *n* (%) | 2 (0.3) | 25 (2.3) |
| Asthma, *n* (%) | 4 (0.5) | 6 (0.5) |
| Chronic rhinitis, *n* (%) | 49 (6.7) | 28 (2.5) |
| Chronic pharyngitis, *n* (%) | 15 (2.1) | 18 (1.6) |
| Chronic cough, *n* (%) | 2 (0.3) | 0 |
| Abnormal liver function, *n* (%) | 20 (2.7) | 53 (4.8) |
| Fatty liver disease, *n* (%) | 44 (6.0) | 68 (6.1) |
| Hepatitis B virus infection, *n* (%) | 29 (4.0) | 30 (2.7) |
| Chronic gastritis, *n* (%) | 16 (2.2) | 14 (1.3) |
| Chronic kidney disease, *n* (%) | 9 (1.2) | 10 (0.9) |
| Diabetes mellitus, *n* (%) | 11 (1.5) | 49 (4.4) |
| Hyperuricemia, *n* (%) | 37 (5.1) | 73 (6.6) |
| Hyperlipidemia, *n* (%) | 15 (2.1) | 53 (4.8) |
| Anemia, *n* (%) | 16 (2.2) | 33 (3.0) |
| Time-to-treatment (hours), median (IQR) | 26.3 (18.5–36.9) | 47.9 (31.8–62.3) |
| Treatment, *n* (%) |  |  |
| Antiviral | 489 (67.0) | 554 (50.1) |
| Placebo | 241 (33.0) | 552 (49.9) |
| COVID-19 severity, *n* (%) |  |  |
| Mild | N/A | 388 (35.1) |
| Moderate | N/A | 718 (64.9) |
| Vaccination status, *n* (%) |  |  |
| Not fully vaccinated | N/A | 45 (4.1) |
| Primary vaccination | N/A | 207 (18.7) |
| Boosted vaccination | N/A | 854 (77.2) |
| Viral load – log10 copies per mL, median (IQR) | 6.10 (5.14–6.85) | 6.45 (5.02–7.67) |

BMI: Body mass index; COVID-19: Coronavirus disease 2019; COPD: Chronic obstructive pulmonary disease; IQR: Interquartile range; N/A: Not applicable.

**Supplementary Table 4: Baseline demographic and clinical characteristics of patients with and without prolonged cough.**

| Items | Influenza | | | COVID-19 | | |
| --- | --- | --- | --- | --- | --- | --- |
|  | Nonprolonged Cough (*N* = 593) | Prolonged Cough (*N* = 101) | *P* value | Nonprolonged Cough (*N* = 875) | Prolonged Cough (*N* = 179) | *P* value |
| Age (years), median (IQR) | 28.0 (20.0–36.0) | 30.0 (22.0–38.0) | 0.22 | 35.0 (28.0–47.0) | 35.0 (28.0–44.0) | 0.43 |
| Female, *n* (%) | 252 (42.5) | 50 (49.5) | 0.23 | 347 (39.7) | 91 (50.8) | 0.01 |
| BMI category, *n* (%) |  |  | 0.90 |  |  | 0.44 |
| BMI <25 kg/m^2^ | 459 (77.4) | 77 (76.2) |  | 562 (64.2) | 121 (67.6) |  |
| BMI ≥25 kg/m^2^ | 134 (22.6) | 24 (23.8) |  | 313 (35.8) | 58 (32.4) |  |
| Smoking status, *n* (%) |  |  | 0.12 |  |  | <0.001 |
| Current smoker | 126 (21.2) | 14 (13.9) |  | 207 (23.7) | 19 (10.6) |  |
| Nonsmoker | 467 (78.8) | 87 (86.1) |  | 668 (76.3) | 160 (89.4) |  |
| Coronary heart disease, *n* (%) | 2 (0.3) | 1 (1.0) | 0.38 | 0 | 0 | 1.00 |
| Hypertension, *n* (%) | 22 (3.7) | 5 (5.0) | 0.58 | 71 (8.1) | 9 (5.0) | 0.21 |
| Chronic lung disease, *n* (%) | 5 (0.8) | 1 (1.0) | 1.00 | 25 (2.9) | 7 (3.9) | 0.47 |
| COPD, *n* (%) | 2 (0.3) | 0 | 1.00 | 21 (2.4) | 3 (1.7) | 0.78 |
| Asthma, *n* (%) | 3 (0.5) | 1 (1.0) | 0.47 | 3 (0.3) | 2 (1.1) | 0.20 |
| Chronic rhinitis, *n* (%) | 39 (6.6) | 8 (7.9) | 0.67 | 25 (2.9) | 2 (1.1) | 0.29 |
| Chronic pharyngitis, *n* (%) | 12 (2.0) | 3 (3.0) | 0.47 | 8 (0.9) | 8 (4.5) | <0.001 |
| Chronic cough, *n* (%) | 0 | 1 (1.0) | 0.15 | 0 | 0 | 1.00 |
| Abnormal liver function, *n* (%) | 16 (2.7) | 3 (3.0) | 0.75 | 48 (5.5) | 5 (2.8) | 0.19 |
| Fatty liver disease, *n* (%) | 31 (5.2) | 9 (8.9) | 0.16 | 55 (6.3) | 11 (6.1) | 1.00 |
| Hepatitis B virus infection, *n* (%) | 23 (3.9) | 4 (4.0) | 1.00 | 24 (2.7) | 6 (3.4) | 0.62 |
| Chronic gastritis, *n* (%) | 10 (1.7) | 4 (4.0) | 0.13 | 10 (1.1) | 4 (2.2) | 0.27 |
| Chronic kidney disease, *n* (%) | 9 (1.5) | 0 | 0.37 | 7 (0.8) | 2 (1.1) | 0.65 |
| Diabetes mellitus, *n* (%) | 8 (1.3) | 1 (1.0) | 1.00 | 44 (5.0) | 5 (2.8) | 0.24 |
| Hyperuricemia, *n* (%) | 35 (5.9) | 2 (2.0) | 0.15 | 58 (6.6) | 13 (7.3) | 0.74 |
| Hyperlipidemia, *n* (%) | 12 (2.0) | 2 (2.0) | 1.00 | 44 (5.0) | 7 (3.9) | 0.70 |
| Anemia, *n* (%) | 15 (2.5) | 1 (1.0) | 0.49 | 23 (2.6) | 8 (4.5) | 0.22 |
| Time-to-treatment (hours), median (IQR) | 26.3 (18.4-36.9) | 28.0 (20.6-40.7) | 0.12 | 47.0 (31.1-62.2) | 52.9 (36.4-64.0) | 0.02 |
| Treatment, *n* (%) |  |  | 0.27 |  |  | 0.44 |
| Antiviral | 406 (68.5) | 63 (62.4) |  | 441 (50.4) | 84 (46.9) |  |
| Placebo | 187 (31.5) | 38 (37.6) |  | 434 (49.6) | 95 (53.1) |  |
| COVID-19 severity, *n* (%) |  |  |  |  |  | 0.36 |
| Mild | N/A | N/A |  | 318 (36.3) | 58 (32.4) |  |
| Moderate | N/A | N/A |  | 557 (63.7) | 121 (67.6) |  |
| Vaccination status, *n* (%) |  |  |  |  |  | 0.63 |
| Not fully vaccinated | N/A | N/A |  | 36 (4.1) | 7 (3.9) |  |
| Primary vaccination | N/A | N/A |  | 159 (18.2) | 38 (21.2) |  |
| Boosted vaccination | N/A | N/A |  | 680 (77.7) | 134 (74.9) |  |
| Viral load – log10 copies per mL, median (IQR) | 6.06 (5.04–6.83) | 6.28 (5.48–7.08) | 0.06 | 6.42 (4.99–7.77) | 6.60 (5.44–7.44) | 0.72 |

BMI: Body mass index; COPD: Chronic obstructive pulmonary disease; COVID-19: Coronavirus disease 2019; IQR: Interquartile range; N/A: Not applicable.

**Supplementary Table 5: Baseline demographic and clinical characteristics of patients with and without** **severe cough.**

| Items | Influenza | | | COVID-19 | | |
| --- | --- | --- | --- | --- | --- | --- |
|  | Nonsevere cough (*N* = 617) | Severe cough (*N* = 113) | *P* value | Nonsevere cough (*N* = 1022) | Severe cough (*N* = 84) | *P* value |
| Age (years), median (IQR) | 28.0 (21.0–36.0) | 28.0 (22.0–38.0) | 0.72 | 36.0 (28.0–47.0) | 32.0 (27.0–40.5) | 0.02 |
| Female, *n* (%) | 255 (41.3) | 61 (54.0) | 0.02 | 416 (40.7) | 41 (48.8) | 0.18 |
| BMI category, *n* (%) |  |  | 0.12 |  |  | 0.20 |
| BMI < 25kg/m^2^ | 469 (76.0) | 94 (83.2) |  | 653 (63.9) | 60 (71.4) |  |
| BMI ≥ 25kg/m^2^ | 148 (24.0) | 19 (16.8) |  | 369 (36.1) | 24 (28.6) |  |
| Smoking status, *n* (%) |  |  | 0.23 |  |  | <0.001 |
| Current smoker | 119 (19.3) | 28 (24.8) |  | 228 (22.3) | 6 (7.1) |  |
| Nonsmoker | 498 (80.7) | 85 (75.2) |  | 794 (77.7) | 78 (92.9) |  |
| Coronary heart disease, *n* (%) | 3 (0.5) | 1 (0.9) | 0.49 | 0 | 0 | 1.00 |
| Hypertension, *n* (%) | 25 (4.1) | 4 (3.5) | 1.00 | 87 (8.5) | 1 (1.2) | 0.01 |
| Chronic lung disease, *n* (%) | 5 (0.8) | 1 (0.9) | 1.00 | 32 (3.1) | 2 (2.4) | 1.00 |
| COPD, *n* (%) | 2 (0.3) | 0 (0) | 1.00 | 24 (2.3) | 1 (1.2) | 1.00 |
| Asthma, *n* (%) | 3 (0.5) | 1 (0.9) | 0.49 | 6 (0.6) | 0 | 1.00 |
| Chronic rhinitis, *n* (%) | 42 (6.8) | 7 (6.2) | 1.00 | 25 (2.4) | 3 (3.6) | 0.47 |
| Chronic pharyngitis, *n* (%) | 12 (1.9) | 3 (2.7) | 0.71 | 16 (1.6) | 2 (2.4) | 0.64 |
| Chronic cough, *n* (%) | 2 (0.3) | 0 | 1.00 | 0 | 0 | 1.00 |
| Abnormal liver function, *n* (%) | 18 (2.9) | 2 (1.8) | 0.75 | 45 (4.4) | 8 (9.5) | 0.06 |
| Fatty liver disease, *n* (%) | 42 (6.8) | 2 (1.8) | 0.05 | 63 (6.2) | 5 (6.0) | 1.00 |
| Hepatitis B virus infection, *n* (%) | 23 (3.7) | 6 (5.3) | 0.43 | 28 (2.7) | 2 (2.4) | 1.00 |
| Chronic gastritis, *n* (%) | 12 (1.9) | 4 (3.5) | 0.29 | 13 (1.3) | 1 (1.2) | 1.00 |
| Chronic kidney disease, *n* (%) | 9 (1.5) | 0 | 0.37 | 9 (0.9) | 1 (1.2) | 0.55 |
| Diabetes mellitus, *n* (%) | 7 (1.1) | 4 (3.5) | 0.07 | 48 (4.7) | 1 (1.2) | 0.17 |
| Hyperuricemia, *n* (%) | 33 (5.3) | 4 (3.5) | 0.64 | 69 (6.8) | 4 (4.8) | 0.65 |
| Hyperlipidemia, *n* (%) | 14 (2.3) | 1 (0.9) | 0.49 | 50 (4.9) | 3 (3.6) | 0.79 |
| Anemia, *n* (%) | 12 (1.9) | 4 (3.5) | 0.29 | 32 (3.1) | 1 (1.2) | 0.51 |
| Time-to-treatment (hours), median (IQR) | 26.1 (17.9–36.4) | 27.7 (22.5–42.2) | 0.03 | 47.7 (30.9–62.2) | 54.3 (39.5–65.2) | 0.02 |
| Treatment, *n* (%) |  |  | 0.79 |  |  | 0.75 |
| Antiviral | 415 (67.3) | 74 (65.5) |  | 510 (49.9) | 44 (52.4) |  |
| Placebo | 202 (32.7) | 39 (34.5) |  | 512 (50.1) | 40 (47.6) |  |
| COVID-19 severity, *n* (%) |  |  |  |  |  | 0.10 |
| Mild | N/A | N/A |  | 366 (35.8) | 22 (26.2) |  |
| Moderate | N/A | N/A |  | 656 (64.2) | 62 (73.8) |  |
| Vaccination status, *n* (%) |  |  |  |  |  | 0.58 |
| Not fully vaccinated | N/A | N/A |  | 41 (4.0) | 4 (4.8) |  |
| Primary vaccination | N/A | N/A |  | 188 (18.4) | 19 (22.6) |  |
| Boosted vaccination | N/A | N/A |  | 793 (77.6) | 61 (72.6) |  |
| Viral load – log10 copies per mL, median (IQR) | 6.12 (5.20–6.85) | 5.95 (4.58–6.79) | 0.10 | 6.42 (5.00–7.63) | 6.95 (6.16–7.77) | 0.10 |

BMI: Body mass index; COPD: Chronic obstructive pulmonary disease; COVID-19: Coronavirus disease 2019; IQR: Interquartile range; N/A: Not applicable.

**Supplementary Table 6: Sensitivity analysis of multivariable logistic regression evaluating risk factors for severe cough in influenza.**

| Items | Original OR | Bootstrap | | Excluding patients with respiratory diseases (*n* = 669) | | |
| --- | --- | --- | --- | --- | --- | --- |
|  |  | Bootstrapped Median | 95% BCa CI  (lower, upper) | OR | 95% CI | *P* value |
| Age | 1.01 | 1.01 | (0.98, 1.03) | 1.01 | (0.99, 1.03) | 0.368 |
| Female | 1.82 | 1.87 | (1.03, 3.57) | 1.95 | (1.08, 3.54) | 0.027 |
| BMI ≥25 kg/m^2^ | 0.79 | 0.81 | (0.40, 1.61) | 0.71 | (0.35, 1.43) | 0.335 |
| Current smoking | 2.05 | 1.95 | (1.07, 3.95) | 2.21 | (1.14, 4.27) | 0.019 |
| Fatty liver disease | 0.38 | 0.36 | (0, 1.52) | 0.45 | (0.10, 2.03) | 0.296 |
| Diabetes mellitus | 2.21 | 2.25 | (0, 12.42) | 2.05 | (0.44, 9.53) | 0.359 |
| Antiviral treatment | 0.83 | 0.83 | (0.51, 1.37) | 0.82 | (0.49, 1.39) | 0.464 |
| Viral load (log copies/mL) | 0.88 | 0.87 | (0.71, 1.08) | 0.86 | (0.72, 1.04) | 0.118 |
| Time-to-treatment (/24 h) | 1.9 | 1.78 | (1.06, 2.94) | 1.95 | (1.13, 3.36) | 0.017 |

BCa: Bias-corrected and accelerated; BMI: Body mass index; CI: Confidence interval; OR: Odds ratio.

**Supplementary Table 7: Sensitivity analysis of multivariate logistic regression showing the risk factors for severe cough in COVID-19.**

| Items | Original OR | Bootstrap | | Excluding Patients with Respiratory Diseases (*n* = 1032) | | |
| --- | --- | --- | --- | --- | --- | --- |
|  |  | Bootstrapped Median | 95% BCa CI  (Lower, Upper) | OR | 95% CI | *P* value |
| Age | 0.96 | 0.96 | (0.93, 0.99) | 0.96 | (0.92, 0.99) | 0.008 |
| Female | 1.01 | 0.99 | (0.50, 2.16) | 0.93 | (0.46, 1.86) | 0.828 |
| BMI ≥25 kg/m^2^ | 0.53 | 0.53 | (0.19, 1.3) | 0.59 | (0.23, 1.33) | 0.224 |
| Current smoking | 0.21 | 0.2 | (0, 0.63) | 0.22 | (0.05, 0.65) | 0.016 |
| Abnormal liver function | 6.86 | 7.31 | (1.14, 20.13) | 7.38 | (2.10, 23.33) | <0.001 |
| Antiviral treatment | 0.97 | 0.98 | (0.45, 2.05) | 0.96 | (0.48, 1.94) | 0.918 |
| COVID-19 severity | 1.49 | 1.53 | (0.58, 3.82) | 1.36 | (0.63, 3.2) | 0.449 |
| Primary vaccination | 0.74 | 0.8 | (0.12, 4359009.92) | 0.77 | (0.16, 5.63) | 0.761 |
| Boosted vaccination | 0.51 | 0.55 | (0.12, 2772635.52) | 0.48 | (0.11, 3.32) | 0.370 |
| Viral load (log copies/mL) | 1.1 | 1.1 | (0.95, 1.27) | 1.12 | (0.94, 1.33) | 0.202 |
| Time-to-treatment (/24 h) | 1.48 | 1.41 | (1.06, 1.85) | 1.41 | (1.05, 1.90) | 0.023 |

BCa: Bias-corrected and accelerated; BMI: Body mass index; COVID-19: Coronavirus disease 2019; CI: Confidence interval; OR: Odds ratio.

**Supplementary Table 8: Sensitivity analysis of multivariate logistic regression evaluating risk factors for prolonged cough in influenza.**

| Items | Original OR | Bootstrap | | Excluding patients with respiratory diseases (*n* = 636) | | |
| --- | --- | --- | --- | --- | --- | --- |
|  |  | Bootstrapped median | 95% BCa CI  (lower, upper) | OR | 95% CI | *P* value |
| Age | 1 | 1.01 | (0.98, 1.03) | 1.01 | (0.98, 1.03) | 0.567 |
| Female | 0.85 | 0.84 | (0.45, 1.53) | 0.74 | (0.40, 1.38) | 0.348 |
| BMI ≥ 25 kg/m^2^ | 0.89 | 0.86 | (0.44, 1.89) | 0.81 | (0.39, 1.68) | 0.580 |
| Current smoking | 0.65 | 0.63 | (0.29, 1.4) | 0.62 | (0.28, 1.35) | 0.229 |
| Antiviral treatment | 0.67 | 0.66 | (0.38, 1.23) | 0.74 | (0.41, 1.31) | 0.302 |
| Viral load (log copies/mL) | 1.24 | 1.25 | (1.01, 1.57) | 1.16 | (0.93, 1.44) | 0.199 |
| Prior severe cough | 2.08 | 2.15 | (0.86, 4.07) | 2.28 | (1.12, 4.66) | 0.023 |
| Time-to-treatment (/24h) | 1.74 | 1.74 | (1.02, 3.03) | 1.84 | (1.05, 3.25) | 0.035 |

BCa: Bias-corrected and accelerated; BMI: Body mass index; CI: Confidence interval; OR: Odds ratio.

**Supplementary Table 9: Sensitivity analysis of multivariate logistic regression evaluating risk factors for prolonged cough in COVID-19.**

| Items | Original OR | Bootstrap | | Excluding Patients with Respiratory Diseases (*n* = 985) | | |
| --- | --- | --- | --- | --- | --- | --- |
|  |  | Bootstrapped Median | 95% BCa CI  (Lower, Upper) | OR | 95% CI | *P* value |
| Age | 1 | 1 | (0.98, 1.02) | 0.99 | (0.97, 1.01) | 0.326 |
| Female | 1.18 | 1.18 | (0.70, 1.98) | 1.25 | (0.77, 2.06) | 0.372 |
| BMI ≥ 25 kg/m^2^ | 1.11 | 1.12 | (0.64, 1.85) | 1.08 | (0.65, 1.78) | 0.763 |
| Current smoking | 0.66 | 0.65 | (0.32, 1.29) | 0.56 | (0.28, 1.07) | 0.094 |
| Chronic pharyngitis | 15.25 | 18.37 | (1.57, 4.20 × 10^7^) | N/A | N/A | N/A |
| Antiviral treatment | 0.65 | 0.64 | (0.39, 1.10) | 0.62 | (0.38, 0.98) | 0.044 |
| COVID-19 severity | 1.33 | 1.34 | (0.81, 2.23) | 1.3 | (0.79, 2.16) | 0.307 |
| Primary vaccination | 1.12 | 1.19 | (0.20, 4018848.22) | 1.18 | (0.28, 8.10) | 0.844 |
| Boosted vaccination | 0.95 | 0.99 | (0.19, 3444498.76) | 1.07 | (0.28, 7.07) | 0.929 |
| Viral load (log copies/mL) | 0.94 | 0.94 | (0.83, 1.07) | 0.95 | (0.85, 1.06) | 0.320 |
| Prior severe cough | 1.7 | 1.72 | (0.25, 5.65) | 2.06 | (0.55, 6.37) | 0.235 |
| Time-to-treatment (/24 h) | 1.28 | 1.28 | (1.04, 1.61) | 1.29 | (1.03, 1.61) | 0.026 |

BCa: Bias-corrected and accelerated; BMI: Body mass index; CI: Confidence interval; COVID-19: Coronavirus disease 2019; OR: Odds ratio.

**Supplementary Table 10: Treatment subgroup analysis assessing the association** **between time-to-treatment and severe or prolonged cough.**

| Explanatory variable | Outcome variable | | Antiviral subgroup**^*^** | Placebo subgroup**^*^** | *P* value for interaction**^†^** |
| --- | --- | --- | --- | --- | --- |
| Time-to-treatment  (unit: day) | Severe cough | Influenza | 1.90 (0.99–3.71) | 2.01 (0.80–5.03) | 0.98 |
|  |  | COVID-19 | 1.52 (0.94–2.49) | 1.46 (0.85–2.54) | 0.91 |
|  | Prolonged cough | Influenza | 1.72 (0.88–3.36) | 1.95 (0.75–5.09) | 0.95 |
|  |  | COVID-19 | 1.36 (1.02–1.82) | 1.18 (0.85–1.64) | 0.52 |

**^*^**Adjusted odds ratio (95% confidence interval) estimated by multivariate logistic regression within each treatment subgroup. **^†^***P* value for the interaction term between treatment group and time-to-treatment in multivariate logistic regression. COVID-19: Coronavirus disease 2019.

**References**

1. Wang Y, Wang H, Liu D, *et al.* Efficacy and safety of single-dose suraxavir marboxil tablet in the treatment of acute uncomplicated influenza in adults: A multi-centre, randomized, double-blind, placebo-controlled phase 2 clinical trial. Clin Microbiol Infect. 2025;31:861–868. doi: 10.1016/j.cmi.2025.01.025.

2. Wang Y, Wang H, Zhang Y, *et al*. Single-dose suraxavir marboxil for acute uncomplicated influenza in adults and adolescents: A multicenter, randomized, double-blind, placebo-controlled phase 3 trial. Nat Med. 2025;31:639–646. doi: 10.1038/s41591-024-03419-3.

3. Cao B, Wang Y, Lu H, *et al.* Oral simnotrelvir for adult patients with mild-to-moderate Covid-19. N Engl J Med 2024;390:230–241. doi: 10.1056/NEJMoa2301425.
